# Ten years for JMCB

**DOI:** 10.1093/jmcb/mjy081

**Published:** 2018-12-22

**Authors:** Jiarui Wu

**Affiliations:** Institute of Biochemistry and Cell Biology, Shanghai Institutes for Biological Sciences, Chinese Academy of Sciences, Shanghai, China

Since *Journal of Molecular Cell Biology* (*JMCB*) was launched in October 2009, it has aimed to be a prestigious journal in the international scientific communities. From the beginning, the editorial board consisted of 77 members from 12 countries all over the world, in which more than half come from the USA. In addition, more than 70% of manuscripts submitted to *JMCB* each year come from overseas authors. We are more than happy to see that *JMCB* has been well recognized by more international scientist groups in the past 10 years.

During my stage as the Editor-in-Chief of *JMCB* since the beginning of 2011, a unique publishing feature has been established for *JMCB*, i.e. each issue, either as a special issue with invited reviews or as a collection for original researches, should focus on a particular topic. Since then, a total of eight special issues have been published, most of which were organized and edited by *JMCB* editorial board members who play active roles in these topic areas. For instance, Drs Hong Ruan and Feng Liu organized a special issue for the adipocyte-specific factor, adiponectin, after two decades of its discovery (Ruan and Liu, 2016). In this issue, Dr Philipp E. Scherer, who was one of the four groups independently identifying adiponectin in 1995, reviewed the history of adiponectin identification and its emerging functional roles in metabolic health and disease (Wang and Scherer, 2016). In 2017, Dr Hua Lu organized a special issue to update the research progress on the MDM2/MDMX–p53 axis, as presented at the 8th International MDM2 Workshop in 2016 and reported in recent literature (Lu, 2017). This issue contained nine well-written review articles covering diverse topics, including either general topics, such as the anatomy of MDM2 and MDMX evolution (Tan et al., 2017), or more specific topics, such as HAUSP regulation of the MDM2/MDMX−p53 axis via deubiquitylation (Tavana and Gu, 2017).

The rest of issues during this period were published as collections involving a number of biological frontiers. The topics varied from basic research to translational research and from biological subjects to interdisciplinary studies. For instance, *JMCB* has recently published two collections on protein science (Wu, 2016a, 2017a), one on developmental biology (Wu, 2016b), one on stem cell biology (Zhou, 2017), two on neuroscience (Wu, 2017b, 2018b), one on cell cycle regulation (Wu, 2018a), and one on non-coding RNA (Wang, 2018) in terms of basic research. Meanwhile, some collections focused on translational research topics, such as complex diseases (Wu, 2016c, d, 2017c) and cancer progression and therapy (Wu, 2016e; Lu, 2018), as well as interdisciplinary studies (Chen, 2017). In this way, *JMCB* could provide enriched and interlinked information with its limited publishing space.

Next year, *JMCB* will be transformed to a fully Open Access journal for monthly publication, which will undoubtedly make the journal more easily to access and capable of publishing more advances in life science. We solicit researchers and readers in all scientific fields to continually support this journal.
